# Revised concept of the genus *Euryporus* Erichson (Coleoptera, Staphylinidae, Staphylininae) and phylogenetic significance of Staphylinini from New Guinea

**DOI:** 10.3897/zookeys.213.3210

**Published:** 2012-08-01

**Authors:** Alexey Solodovnikov

**Affiliations:** 1 Zoological Museum, Natural History Museum of Denmark, Universitetsparken 15, Copenhagen 2100, Denmark

**Keywords:** *Euryporus*, *Tympanophorus*, *Hesperus*, Quediina, Anisolinina, Philonthina, Staphylinini, New Guinea

## Abstract

The Staphylinini rove beetle genus *Euryporus* Erichson from the subtribe Quediina is restricted to include only three species from the Western Palearctic region: *Euryporus picipes* (Paykull, 1800), *Euryporus aeneiventris* (Lucas, 1846), and *Euryporus princeps* Wollaston, 1864. *Euryporus argentatus* Fauvel, 1881, *Euryporus warisensis* Last, 1987 and *Euryporus multicavus* Last, 1980, which do not even belong to the subtribe Quediina, are excluded fromthe genus. Of these, two were transferred to different genera: *Tympanophorus argentatus* (Fauvel, 1881), **comb. nov.**, from Sumatra;and *Hesperus warisensis* (Last, 1987), **comb. nov.**,from New Guinea. “*Euryporus” multicavus* could not be placed to any of the described genera of Staphylinini and is left as *incertae sedis* pending a broader study of the relevant fauna of this tribe in New Guinea and adjacent regions. The taxonomic history of *Euryporus* is reviewed, and an updated diagnosis of this genus is provided.

## Introduction

An abundance of large and polyphyletic, poorly defined genera is a drawback of the current classification of the hyper-diverse rove beetle tribe Staphylinini (e.g., “*Quedius*-complex” discussed in Solodovnikov 2006). By including numerous unrelated species together, such “genera” inhibit species discovery and taxonomic revisions, and they introduce “noise” in any evolutionary study of rove beetles. However, a number of monobasic or species-poor genera of Staphylinini suffer from the flawed definition too.

**Figure 1.**
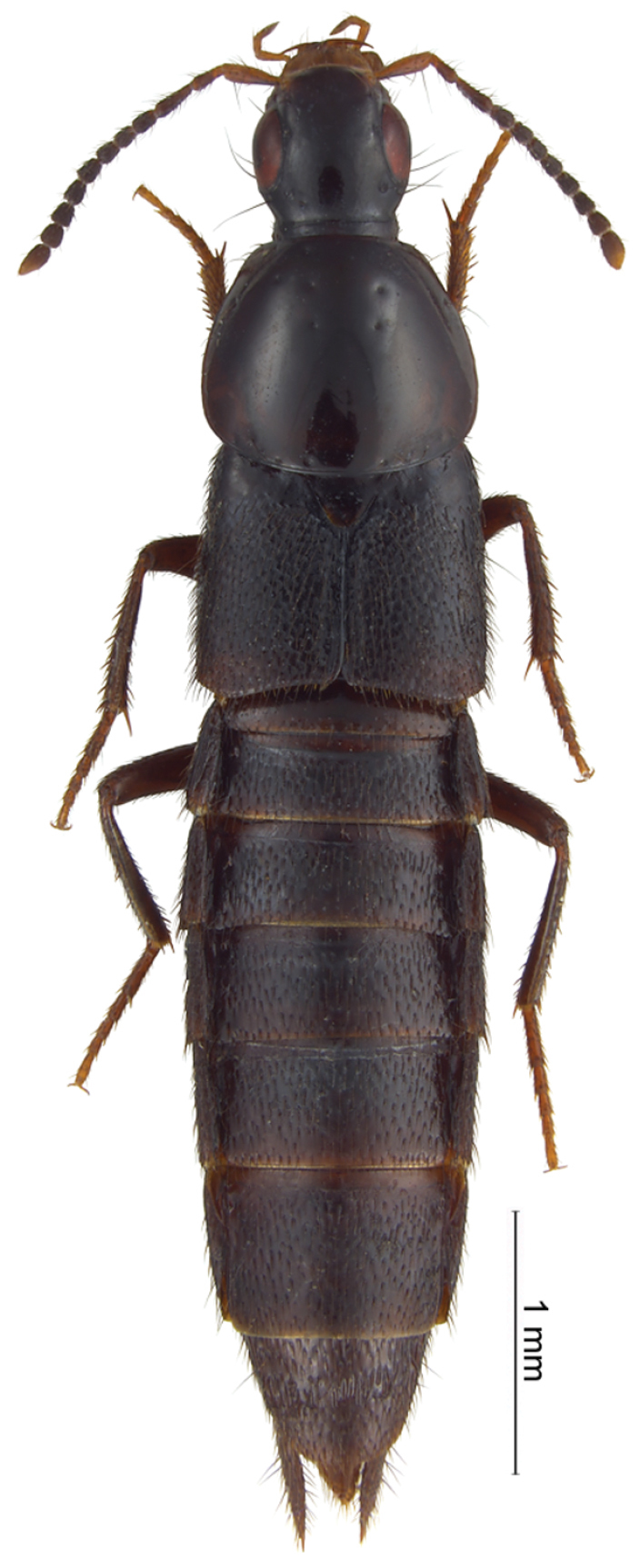
*Euryporus picipes*, habitus.

**Figure 2.**
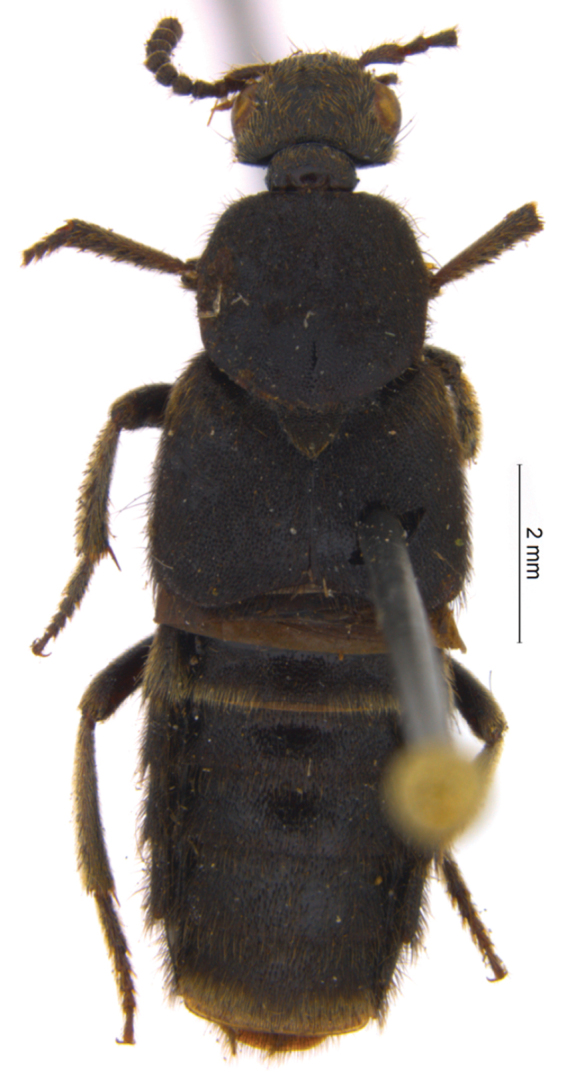
*Tympanophorus argentatus*, holotype, habitus.

**Figures 3–7.**
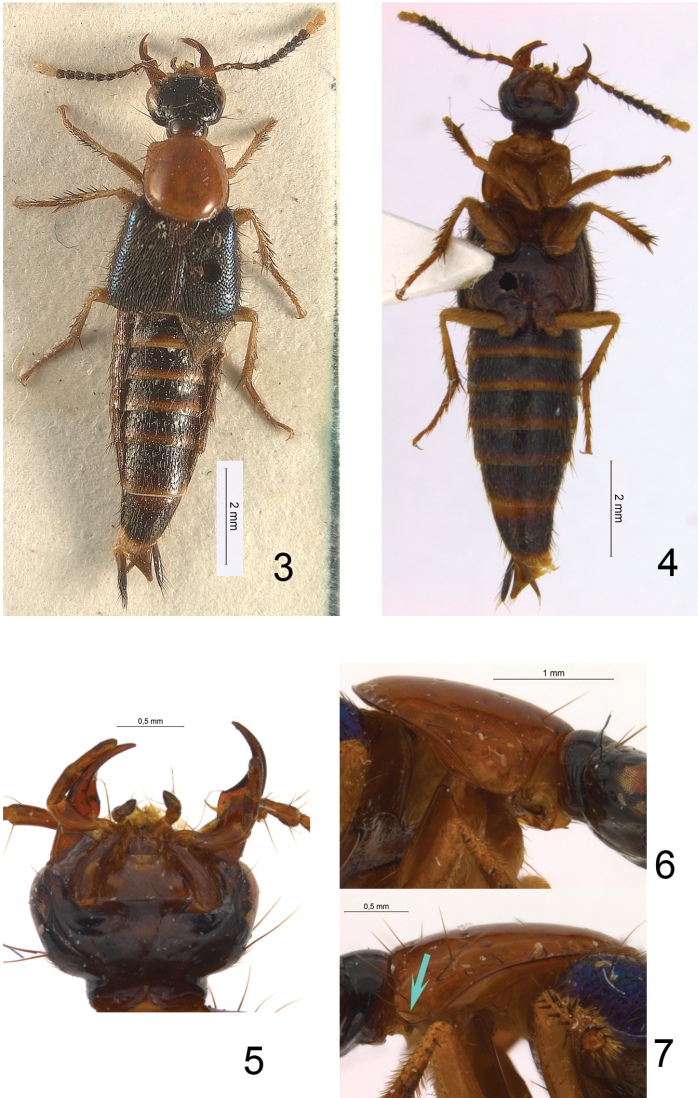
*Hesperus warisensis*, holotype: **3** habitus **4** body in ventral view **5** head in ventral view **6** right side of pronotum in lateral view **7** left side of pronotum in lateral view. Blue arrow shows “fake” superior line of pronotum.

**Figures 8–11.**
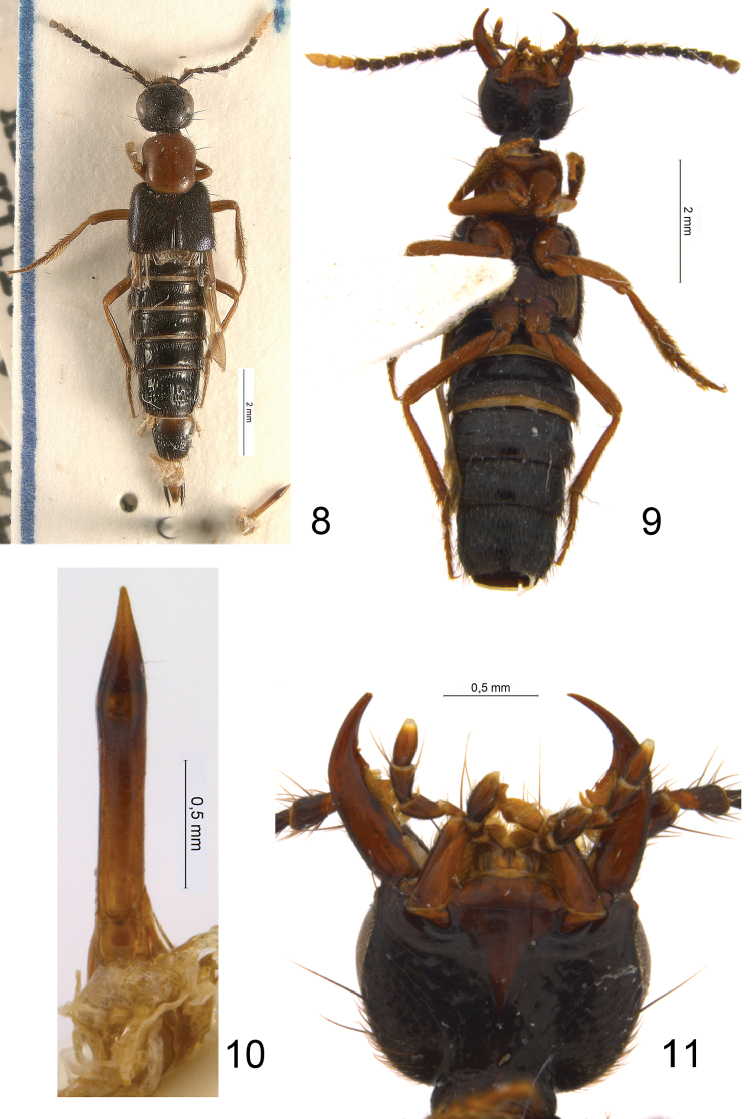
*“Euryporus” multicavus*, paratype: **8** habitus **9** body in ventral view **10** aedeagus in parameral view **11** head in ventral view.

One such small genus that nevertheless turned out to be a taxonomic “waste basket” is *Euryporus* Erichson, 1839 from the subtribe Quediina. Prior to this paper *Euryporus* comprised three well-known species from the Western Palearctic region (*Euryporus picipes* (Paykull, 1800) (Fig. 1), *Euryporus aeneiventris* (Lucas, 1846), and *Euryporus princeps* Wollaston, 1864), and three poorly known “exotic” species: *Euryporus argentatus* Fauvel, 1881 from Sumatra (Fig. 2), as well as *Euryporus warisensis* Last, 1987 (Figs 3–7) and *Euryporus multicavus* Last, 1980 (Figs 8–11) from New Guinea. Poor descriptions of these “exotic” species coupled with the unusual disjunct distribution of the genus cast strong doubts on the monophyly of *Euryporus* and triggered this study.

Examination of the relevant types made the misplacement of all three “exotic” species in *Euryporus* immediately obvious. But while the correct identity of *Euryporus argentatus* and *Euryporus warisensis* as members ofthe genera *Tympanophorus* Nordmann, 1837 and *Hesperus* Fauvel, 1874, respectively, also became clear, proper classification of *Euryporus multicavus* faced a problem of poor generic limits in the subtribes Philonthina and Anisolinina, and even a problem of blurred limit between these subtribes (Schillhammer 2004). In such circumstances, a broader phylogenetic analysis embracing relevant lineages from these and related subtribes of Staphylinini would be required. For the poorly known fauna of New Guinea and adjacent regions such analysis was impossible without prior extensive taxonomic study of many species, which was far beyond the scope and goals of this paper. Therefore, *Euryporus multicavus* is explicitly removed from *Euryporus* but left as *incertae sedis* within Staphylinini pending further study.

## Material and methods

The paper is based on the material from the following institutions:

BPBM Bernice P. Bishop Museum, Honolulu (S. Myers)

HNHM Hungarian Natural History Museum, Budapest (G. Makranczy)

MMUE Manchester Museum, the University of Manchester (D. Logunov)

NCBN Netherlands Centre for Biodiversity Naturalis, the Netherlands (M.E. Gassó Miracle and A. van Assen)

Labels of the examined types are quoted verbatim; data from each label are separated by a slash [/].

Photos in Figs 3 and 8 were taken by the author with an MP-E 65 mm lens for Canon EOS 40D; those in Figs 2, 4–7, and 9–11 were taken by Ken Puliafico (Copenhagen) with a Leica DFC 420 camera attached to a Leica MZ16A microscope with the aid of Leica Application Suite (Leica Microsystems, 2003-2007) and Automontage Pro (Synoptics Ltd, 1997–2004). The photo in Fig. 1 was produced and kindly provided by Harald Schillhammer (Vienna).

Correspondence of the locality names from old collection labels to modern toponyms was checked with the on-line resource (http://isodp.hof-university.de/fuzzyg/query/ ).

## Genus Euryporus

Erichson, 1839

http://species-id.net/wiki/Euryporus

### Type species.

*Oxyporus picipes* Paykull, 1800 (fig. 1).

### Taxonomic history.

The rove beetle genus *Euryporus* Erichson, 1839 was described by Nordmann (1837) as *Pelecyphorus* to include one European species *Euryporus picipes* (Paykull, 1800) (Fig. 1). Since *Pelecyphorus* Nordmann, 1837 (nec *Pelecyphorus* Dejean, 1834) was a preoccupied name, Erichson (1839) replaced it with *Euryporus* and described the second species in the genus, *Euryporus puncticollis* from North America (Erichson 1840). Soon, *Euryporus aeneiventris* Lucas, 1846 and *Euryporus princeps* Wollaston, 1864, both from the West Palearctic region were added (Lucas 1846; Wollaston 1864). Later Fauvel (1881, 1884) described *Euryporus argentatus* Fauvel, 1881 and *Euryporus flavipes* Fauvel, 1884, both from Sumatra. On the contrary, two species were removed from the genus: Sharp (1884) transferred Erichson's *Euryporus puncticollis* to the genus *Tympanophorus* Nordmann, 1837, while Fauvel (1895) erected a new genus *Pammegus* (now with twelve species, in the subtribe Anisolinina) for his own species *Euryporus flavipes*. Finally, Last (1980, 1987) described two more species in *Euryporus*: *Euryporus multicavus* Last, 1980 and *Euryporus warisensis* Last, 1987, both from Papua New Guinea.

As a result, the genus *Euryporus* included six species before this study (e.g., Herman 2001). Of them the type species *Euryporus picipes* and two other West Palearctic species, *Euryporus aeneiventris*, and *Euryporus princeps*, are very similar to each other and rather well-known (e.g., Coiffait 1978, Assing and Schülke 2012). Examination of the type material for the “exotic” *Euryporus argentatus*, *Euryporus multicavus* and *Euryporus warisensis* led to their exclusion from *Euryporus* as explained below.

### Updated diagnosis, composition and phylogenetic relationships.

Without the excluded taxa (see below), *Euryporus* comprises three species very similar to each other: *Euryporus picipes* (Paykull, 1800) widely distributed in Europe (Fig. 1); the West Mediterranean *Euryporus aeneiventris* Lucas, 1846; and *Euryporus princeps* Wollaston, 1864, endemic to the Canary Islands. Male genitalia of all species were illustrated in Coiffait (1978).

Among other genera of the subtribe Quediina, *Euryporus* can be distinguished by the following combination of characters: fully developed infraorbital ridges; mandibles elongate with broad basal part but narrow and sharp apical portion; last segment of maxillary palps fusiform, slightly setose; last segment of labial palps enlarged, apically obliquely truncated, densely setose; first antennal segment elongate, as long as second and third antennal segments together; anterior tarsi narrow, not enlarged in both sexes; apical margin of abdominal sternite VIII in both sexes concave, in male without median incision. Other recent descriptions and synopses of the genus can be found in Coiffait (1978) and Assing and Schülke (2012).

For phylogenetic purposes adult (Solodovnikov 2006; Solodovnikov and Schomann 2009) and larval (Pietrykowska-Tudruj et al. 2011) morphology of *Euryporus picipes* was scored in those character matrixes. The adult-based analysis (Solodovnikov and Schomann 2009) placed *Euryporus* in the subtribe Quediina (in the restricted sense of Chatzimanolis et al. 2010). Within Quediina, it may be related to the lineage formed by the genera *Anaquedius* Casey, 1915, *Hemiquedius* Casey, 1915, *Anchocerus* Fauvel, 1905, *Australotarsius* Solodovnikov et Newton, 2009, and *Acylophorus* Nordmann, 1837 (Solodovnikov and Schomann 2009; but see additional remarks about alternative hypotheses in Solodovnikov and Newton 2009). Although *Euryporus* was not included in the molecular study of Chatzimanolis et al. (2010) because of unavailable DNA-quality material, the above mentioned lineage was recovered as monophyletic in the Bayesian analysis of that study. The larvae-based analysis (Pietrykowska-Tudruj et al. 2012) was inconclusive as far as sister relationships of *Euryporus* is concerned.

## Species excluded from *Euryporus*

### Tympanophorus argentatus

(Fauvel, 1881) comb. n.

http://species-id.net/wiki/Tympanophorus_argentatus

Figure 2

#### Type material examined.

**Indonesia:** Holotype, female, “*Euryporus argentatus* Fvl. [in Fauvel's handwriting] / Suon Exp. Moeara Laboe 11/77 [circle label]/ Museum Leiden *Euryporus argentatus* det. Fauv. [pre-printed, partly handwritten curatorial label]/ *argentatus* Fauvel n. sp. [handwritten label]/ Holotype *Euryporus argentatus* Fauv. revised by A. Solodovnikov 2012 [red label]/*Tympanophorus argentatus* (Fauvel) A. Solodovnikov det. 2012” (NCBN).

*Comments*. In the original description of *Euryporus argentatus*, Fauvel (1881) clearly mentioned a single type specimen from “Moeara Laboe” [= Moearalaboeh, now Propinsi Jambi, Indonesia, 1°29'0"S, 101°3'0"E]. Based on the habitus (Fig. 2) and other diagnostic characters, the holotypeand other specimens of *Euryporus argentatus* from the collection of NCBN are conspecific and can be clearly identified as a species of the genus *Tympanophorus* Nordmann, 1837. With the possible exception of *Tympanophorus schenklingi* Bernhauer, 1912 from the Afrotropical region, *Tympanophorus* (e.g., illustrated redescription in Naomi 1983) is monophyletic (Schillhammer 2004).

It is noteworthy that long after the description of *Euryporus argentatus*, Fauvel (1902) did recognize the correct affiliation of that species. In a short note on page 42 he mentioned “*Tympanophorus argentatus* Fvl. (*rugosus* Waterh.)”, apparently meaning a synonymy of his species with *Tympanophorus rugosus* (C. Waterhouse, 1884). This so vaguely annotated transfer of *Euryporus argentatus* to *Tympanophorus* was overlooked by later authors. For example Herman (2001) lists both *Euryporus argentatus* Fauvel, 1881 as a valid species and “*Tympanophorus argentatus* Fauvel”, erroneously, as *nomen nudum*. Synonymy of *Tympanophorus argentatus* (Fauvel, 1881) and T. *rugosus* (C. Waterhouse, 1884) remains to be verified.

### Hesperus warisensis

(Last, 1987) comb. n.

http://species-id.net/wiki/Hesperus_warisensis

Figures 3–7

#### Type material examined.

**Papua New Guinea:** Holotype, female, “Holotype [red circular label]/ New Guinea Neth. Waris, S. of Hollandia, 450–500 m, VIII-16-23-1959/ T.C. Maa collector Bishop/ *Euryporus warisensis* sp. n. H.R. Last det., Holotype [H.R. Last's label]/ *Hesperus warisensis* (Last) A. Solodovnikov det. 2012” (BPBM).

Although *Euryporus warisensis* is strikingly different from the Palearctic *Euryporus* (cf. Figs 1 and 3), Last (1987) did not provide any justification for his generic placement. Based on the structure of head sutures (rudimentary infraorbital ridges, Fig. 5; present dorsal basal ridge on the neck), prothorax (laterally visible hypomera; superior marginal line turning downwards before anterior angles of pronotum, Fig. 6); anterior angles of pronotum not strongly protruding over anterior margin of prothorax), legs (lacking empodial setae) and other characters, *Euryporus warisensis* is clearly not congeneric with *Euryporus* and in fact belongs to the subtribe Philonthina.

Because of its rather elongate mandibles and maxillary palps (Fig. 5), as well as habitus resemblance, *Euryporus warisensis* could be associated with some species of *Hesperus* from New Guinea like *Hesperus raynori* Last, 1987 and others. As pointed out in Schillhammer (2002) about *Hesperus* [“…this genus is a dumping ground for species matching a particular set of characters which can hardly suffice to justify a monogeneric treatment”], and demonstrated in the phylogenetic analysis (Li and Zhou 2011), this genusis not a monophyletic group and needs a revision. In such circumstances placement of *Euryporus warisensis* in *Hesperus* is a practical solution pending further study. As far as I am aware (and personal communication of H. Schillhammer), the enlarged apical labial palpomeres of *Euryporus warisensis* easily distinguish this species from any other known species of *Hesperus*.

It is noteworthy that on the left side of the pronotum (Fig. 7) the holotype of *Hesperus warisensis* displays a “fake” superior line extended towards anterior angles of pronotum, while the right side has no such structure (Fig. 6). Presumably, the left side of the pronotum in the holotype displays a slight teratology.

### Euryporus multicavus

Last, 1980 non Euryporus, Staphylinini incertae sedis

http://species-id.net/wiki/Euryporus_multicavus

Figures 8 –11

#### Type material examined.

**Papua New Guinea:** Holotype, male, “New Guinea SE Kiunga, 1.VIII. 1969/ No. NGK-R. 1 leg. Dr. Ballogh/ Holotypus 1980 male [symbol] *Euryporus multicosus* [sic!] Last [standard HNHM curatorial label] / *Euryporus multicosus* [sic!] sp. n. H.R. Last det., Type male [symbol] [H.R. Last's label]” (HNHM); paratype, male, “New Guinea SE Kiunga, 23.VII-2.VIII.1969/ No. NGK-B.3. leg. Dr. Ballogh/ *Euryporus multicavus* sp. n. H.R. Last det., Paratype [H.R. Last's label]/ Staphylinini genus nov.? A. Solodovnikov det. 2012” (MMUE).

*Comments*. As in the above described case, *Euryporus multicavus* is strikingly different from the Palearctic *Euryporus* in habitus (cf. Figs 1 and 8), but Last (1980) did not explain why his species was assigned to that genus. Based on the structure of head (rudimentary infraorbital ridges (Fig. 11); present dorsal basal ridge on the neck), prothorax (superior marginal line inflected inwards under anterior angles of pronotum; pronotal hypomera visible from lateral view; anterior angles of pronotum not strongly protruding over anterior margin of pronthorax), legs (lacking empodial setae) and other characters, it is clear that *Euryporus multicavus* is not congeneric with *Euryporus* and even does not belong to the subtribe Quediina. On the other hand, the combination of characters of that species does not allow its unambiguous placement in any of the currently recognized subtribes of Staphylinini.

Because of the short and stout labial palps with dilated last segment, shape of the mandibles (Fig. 11), strongly foveate surface of the apical abdominal segments, and the overall habitus (Fig. 8)remotely resembling *Tympanophorus*, I assume that “*Euryporus” multicavus* is phylogenetically close to the *Tympanophorus*-lineage of the subtribe Anisolinina (as defined in Schillhammer 2004). But the absence of the elevated ridge on the mesosternum, absence of empodial setae, sexually dimorphic sternite VII (with slight medio-apical incision in male) and strongly reduced paramere of the aedeagus (Fig. 10), cast doubts on such affinity. At least the absence of empodial setae and extremely reduced paramere of the aedeagus are shared by “*Euryporus” multicavus* with several species from New Guinea described in the genera *Philonthus* and *Hesperus*. But, except *Hesperus warisensis* moved to that genus here, none of those species have robust and dilated labial palpi, and all of them differ from “*Euryporus” multicavus* in other details. It is possible that “*Euryporus” multicavus* represents a new genus whose description must be postponed until a more inclusive phylogenetic study of relevant lineages is performed. Such study should be based not only on an extensive taxonomic revision of the hitherto poorly described relevant species but also include additional material from the collections of Staphylinini from New Guinea and adjacent regions, which I am aware of and which have remained largely untouched by modern workers.

## Supplementary Material

XML Treatment for Euryporus

XML Treatment for Tympanophorus argentatus

XML Treatment for Hesperus warisensis

XML Treatment for Euryporus multicavus

## References

[B1] Assing V Schülke M (2012) Freude–Harde–Lohse–Klausnitzer – Die Käfer Mitteleuropas. Band 4. Staphylinidae I. Zweite neubearbeitete Auflage. Spektrum Akademischer Verlag, Heidelberg.

[B2] Chatzimanolis S Cohen I Schomann A Solodovnikov A (2010) Molecular phylogeny of the mega-diverse rove beetle tribe Staphylinini (Insecta, Coleoptera, Staphylinidae). Zoologica Scripta 39 (5): 436-449. doi: 10.1111/j.1463-6409.2010.00438.x

[B3] Coiffait H (1978) Coléoptères Staphylinidae de la région paléarctique occidentale. Nouvelle Revue d'Entomologie 8 (4): 1-364.

[B4] Erichson WF (1839) Die Käfer der Mark Brandenburg. FH Morin, Berlin, 385–740.

[B5] Erichson WF (1840) Genera et species Staphylinorum insectorum coleopterorum familiae. FH Morin, Berlin, 40–954.

[B6] Fauvel A (1881) Duae novae Staphylinidae ex India orientali (Sumatra). Notes from the Leyden Museum 3: 163-165.

[B7] Fauvel A (1884) Description of a new species of the coleopterous family Staphylinidae. Notes from the Leyden Museum 6: 241-242.

[B8] Fauvel A (1895) Staphylinides nouveaux de l'Inde et de la Malaisie. Revue d'Entomologie 14: 180–286.

[B9] Fauvel A (1902) Bibliographie. Zur Staphyliniden-Fauna von Ceylan, von Dr. Max Bernhauer. Revue d'Entomologie 21: 40-43.

[B10] Herman LH (2001) Catalog of the Staphylinidae (Insecta: Coleoptera). 1758 to the end of the second millennium. IV. Staphylinine group. Bulletin of the American Museum of Natural History 265: 2441-3020.

[B11] Last HR (1980) Records of New Guinea Staphylinidae (Coleoptera) in the Hungarian Natural History Museum. Annales Historico-Naturales Musei Nationalis Hungarici 72: 139-161.

[B12] Last HR (1987) Staphylinidae from Papua New Guinea in the collections of Bernice P. Bishop Museum Honolulu, Hawaii (Insecta, Coleoptera). Entomologische Abhandlungen 51 (3): 25-56.

[B13] Li L Zhou HZ (2011) Revision and phylogenetic assessment of the rove beetle genus *Pseudohesperus* Hayashi, with broad reference to the subtribe Philonthina (Coleoptera: Staphylinidae: Staphylinini) Zoological Journal of the Linnean Society 163(3): 679–722. doi: 10.1111/j.1096-3642.2011.00731.x

[B14] Lucas PH (1846) Histoire naturelle des animaux articulé. Deuxième partie. Insectes. In: Exploration scientifique de l'Algérie…. Sciences Physiques. Zoologie. 2, Gouvernement Nationale, Paris, 590 pp.

[B15] Naomi S-I (1983) Revision of the subtribe Xanthopygina (Coleoptera, Staphylinidae) of Japan II. Kontyũ 51 (1): 47-55.

[B16] Nordmann A von (1837) Symbolae ad monographiam staphylinorum. Ex Academiae Caesareae Scientarum 4, Academiae Caesareae Scientarum, Petropoli, 1–167.

[B17] Pietrykowska-Tudruj E Staniec B Solodovnikov A (2011) Discovery of the *Quedius antipodum* Sharp larva from New Zealand: phylogenetic test of larval morphology for Staphylinini at the intratribal level (Coleoptera: Staphylinidae). Systematic Entomology 37 (2): 360-378. doi: 10.1111/j.1365-3113.2011.00612.x

[B18] Schillhammer H (2002) Three new Oriental species of *Hesperus* Fauvel (Coleoptera: Staphylinidae). Koleopterologische Rundschau 72: 127-135.

[B19] Schillhammer H (2004) Critical notes on the subtribe Anisolinina with descriptions of nine new species (Coleoptera: Staphylinidae: Staphylininae). Koleopterologische Rundschau 74: 251-277.

[B20] Sharp DS (1884) Staphylinidae. In: Porter RH (Ed.). Biologia Centrali-Americana: Insecta. Coleoptera. 1(2). Taylor & Francis, London: 537-672.

[B21] Solodovnikov AY (2006) Revision and phylogenetic assessment of *Afroquedius* gen. nov. from South Africa: toward new concepts of the genus *Quedius*, subtribe Quediina and reclassification of the tribe Staphylinini (Coleoptera: Staphylinidae: Staphylininae). Annals of the Entomological Society of America 99 (6): 1064-1084. doi: 10.1603/0013-8746(2006)99[1064:RAPAOA]2.0.CO;2

[B22] Solodovnikov AY Newton AF (2009) *Australotarsius* – a new genus of the rove beetle tribe Staphylinini from Australia (Coleoptera: Staphylinidae: Staphylininae) Zootaxa2033: 49–57. doi: 10.1111/j.1365-3113.2008.00468.x

[B23] Solodovnikov A Schomann A (2009) Revised systematics and biogeography of “Quediina” of Subsaharan Africa: new phylogenetic insights into the rove beetle tribe Staphylinini (Coleoptera: Staphylinidae). Systematic Entomology 34: 443-446.

[B24] Wollaston TV (1864) Catalogue of the coleopterious insects of the Canaries in the collection of the British Museum. British Museum, London, 648 pp.

